# Alveolar macrophage-fibroblast crosstalk includes a feedforward arginase 1/IL-6 circuit implicated in pulmonary fibrosis

**DOI:** 10.1172/JCI200729

**Published:** 2025-11-03

**Authors:** G.R. Scott Budinger

**Affiliations:** Division of Pulmonary and Critical Care Medicine, and Simpson Querrey Lung Institute for Translational Science (SQ LIFTS), Northwestern University Feinberg School of Medicine, Chicago, Illinois, USA.

Alveolar macrophages are implicated in the development of lung fibrosis in mice after the administration of bleomycin (1). Their role in patients with interstitial lung disease (ILD), however, has been controversial; although clinical trials of nonselective antiinflammatory therapies, most importantly corticosteroids, have failed to show benefit in patients with idiopathic pulmonary fibrosis (IPF) (2), clinical trials of antiinflammatory therapies have shown benefit in patients with connective tissue disease–associated ILD (3).

In both normal and fibrotic human lungs, the receptor for colony stimulating factor 1 (CSF1R) is exclusively expressed by alveolar and interstitial macrophages and dendritic cells. Recently, Wolff et al. published the results of a phase II clinical trial of axatilimab, an inhibitory antibody targeting the CSF1R, in patients with multiorgan fibrosis secondary to graft-versus-host disease (GVHD) refractory to other therapies (4). The results of the trial were impressive, with an overall response rate of 74%. Remarkably, 26% of patients showed reversal of established fibrosis in the skin and 47% showed improvement in lung fibrosis as measured by symptoms or pulmonary function tests. These data led to rapid FDA approval of axatilimab for treatment of refractory GVHD and initiation of the MAXPIRe trial of axatilimab in patients with IPF (ClinicalTrials.gov NCT06132256). If confirmed in larger trials, axatilimab would be the only example of a therapy that reverses established lung fibrosis in humans. These findings create an urgent need to understand the mechanistic link between CSF1R-expressing cells and the disordered epithelial repair and fibroblast activation implicated in lung fibrosis.

In this issue of the *JCI*, Yadav et al. describe one such mechanism (5). They build upon work in mice suggesting that inhibiting CSF1R interrupts reciprocal signaling loops between alveolar macrophages and fibroblasts to reverse established fibrosis induced by asbestos administration (6). Yadav and colleagues found that monocyte-derived alveolar macrophages expressed arginase 1 (ARG1), the enzyme responsible for production of ornithine, which augments proline and collagen synthesis by fibroblasts. Fibroblasts in turn produced IL-6 to promote ARG1 expression in CSFR1^+^ alveolar macrophages in response to signaling by extracellular ATP, thus underscoring the reciprocal interactions between these macrophages and fibroblasts in fibrotic disease. Using a rigorous experimental design, Yadav et al. used high-depth spatial transcriptomics to show that human ARG1 was not detected in monocyte-derived alveolar macrophages. Instead, ARG1 was readily and robustly detected in human neutrophils, reflecting an important discrepancy between humans and mouse models.

The results reported by Yadav et al. (5) are important in themselves and raise critical questions for the field. First, which populations of CSF1R-expressing cells in the fibrotic lung directly mediate fibrosis? Second, do these cells cause fibrosis through direct interactions with fibroblasts in the interstitium? Third, what are the limitations of murine models of lung fibrosis, and can inflammatory cells be successfully incorporated into existing human platforms (e.g., precision cut lung slices and organoids) to better model human lung fibrosis? Given the possibility of reversing established lung fibrosis in some patients, excitement around these questions is high.

## Version 1 11/03/2025

Electronic publication
